# Assessment of Drivers of Antimicrobial Usage in Poultry Farms in the Mekong Delta of Vietnam: A Combined Participatory Epidemiology and Q-Sorting Approach

**DOI:** 10.3389/fvets.2019.00084

**Published:** 2019-03-25

**Authors:** Dinh Bao Truong, Hoang Phu Doan, Vinh Khanh Doan Tran, Van Cuong Nguyen, Tuan Kiet Bach, Chalalai Rueanghiran, Aurélie Binot, Flavie L. Goutard, Guy Thwaites, Juan Carrique-Mas, Jonathan Rushton

**Affiliations:** ^1^Oxford University Clinical Research Unit, Ho Chi Minh, Vietnam; ^2^Faculty of Animal Science and Veterinary Medicine, Nong Lam University, Ho Chi Minh, Vietnam; ^3^Faculty Veterinary Medicine, Kasetsart University, Bangkok, Thailand; ^4^Sub Department of Animal Health and Production, Cao Lãnh, Vietnam; ^5^Department of Veterinary Public Health, Faculty of Veterinary Medicine, Kasetsart University, Bangkok, Thailand; ^6^ASTRE, CIRAD, INRA, University Montpellier, Montpellier, France; ^7^Centre for Tropical Medicine and Global Health, Oxford University, Oxford, United Kingdom; ^8^Institute of Infection and Global Health, University of Liverpool, Liverpool, United Kingdom

**Keywords:** antimicrobial usage, Q-sorting, participatory epidemiology, farmers' attitude, discourse

## Abstract

In the Mekong Delta of Vietnam, poultry farmers use high amounts of antimicrobials, but little is known about the drivers that influence this usage. We aimed to identify these drivers using a novel approach that combined participatory epidemiology (PE) and Q-sorting (a methodology that allows the analysis of the subjectivity of individuals facing a common phenomenon). A total of 26 semi-structured collective interviews were conducted with 125 farmers representative of the most common farming systems in the area (chickens, meat ducks, and mobile grazing ducks), as well as with 73 farmers' advisors [veterinarians, veterinary drug shop owners, and government veterinarians/commune animal health workers (CAHWs)] in five districts of Dong Thap province (Mekong Delta). Through these interviews, 46 statements related to the antimicrobials' perceived reliability, costs, and impact on flock health were created. These statements were then investigated on 54 individuals (28 farmers and 26 farmers' advisors) using Q-sorting interviews. Farmers generally indicated a higher propensity for antimicrobial usage (AMU) should their flocks encounter bacterial infections (75.0–78.6%) compared with viral infections (8.3–66.7%). The most trusted sources of advice to farmers were, in decreasing order: government veterinarian/CAHWs, their own knowledge/experience, veterinary drug shop owners, and sales persons from pharmaceutical and feed companies. The highest peak of AMU took place in the early phase of the production cycle. Farmers and their advisors showed considerable heterogeneity of attitudes with regards to AMU, with, respectively, four and three discourses representing their views on AMU. Overall, farmers regarded the cost of AMU cheaper than other disease management practices implemented on their farms. However, they also believed that even though these measures were more expensive, they would also lead to more effective disease prevention. A key recommendation from this finding would be for the veterinary authorities to implement long-term sustainable training programs aiming at reducing farmers' reliance on antimicrobials.

## Introduction

The misuse (over- and under-use) of antimicrobials in animal production is one of the contributing factors of the global emergency of antimicrobial resistance (AMR) (1). Levels of antimicrobial usage (AMU) in low- and middle-income countries (LMICs) are particularly high (2), and are expected to increase markedly over coming years due to intensification of animal production and increased demand for animal protein (3, 4). In the Mekong Delta of Vietnam farmers typically use large amounts of antimicrobials to raise poultry, and a high incidence of disease has been reported in chicken flocks (5). A recent study showed that, on average, 470 mg antimicrobial compounds were used to produce one meat chicken, and most of the AMU was aimed at preventing, rather than treating disease (6, 7). A survey conducted in Cambodia on small-scale pig farms showed that the farmer's own judgment was the most important determinant associated with AMU (8). Another survey on small- and medium-scale pig farms in northeastern Thailand indicated that two thirds (68%) of small-scale farmers decided themselves whether or not to give antimicrobials to their animals, whereas all medium-scale farmers discussed antimicrobial treatments with a veterinarian (9). When using antimicrobials to treat disease, European pig farmers were more interested in the short-term impact on their herds' health than in the AMR “side effects” (10). A study on Vietnamese poultry farms confirmed that, from the farmers' point of view, the main target is to maintain the highest possible number of birds alive until end of production (11). A study of poultry farmers in the Mekong Delta found that the farmers' sources of advice were: drug sellers (56%), followed by the district veterinarian (18%), and farmers colleagues (12%) (6). However, there is a gap in knowledge on the farmers' perception of the antimicrobials' effectiveness and the socio-economic factors driving AMU in the Mekong Delta of Vietnam. This knowledge is critical for the design and implementation of intervention strategies.

The study used two well-documented methods to fill this knowledge gap: Participatory Epidemiology and Q-sorting. Participatory epidemiology (PE) is the systematic use of participatory approaches and methods to improve the understanding of diseases and options for animal disease control. PE involves communities to define and prioritize animal health problems, and to improve veterinary service delivery, control and/or surveillance of diseases (12). PE draws on widely accepted techniques of participatory rural appraisal, ethno-veterinary surveys, and qualitative epidemiology (13). Q-sorting is a qualitative method used to analyse the subjective perception of individuals in relation to a particular situation or phenomenon. Q-sorting helps identify trends and convergences of opinions (14), and has been used in a wide variety of research areas, such as political subjectivity (14), public health (15, 16), veterinary science (17), and rural sociology (18, 19).

Specific objectives of the study were: (a) to identify the relative frequency of disease in flocks and the farmers' propensity for using antimicrobials should disease appear; (b) to identify the timing of antimicrobial administration in relation to the amounts used; (c) to define the sources of advice and procurement of antimicrobials to farmers; (d) to identify farmers' positive and negative opinions on AMU; and (e) to investigate socio-economic factors influencing farmers' attitudes on AMU.

## Materials and Methods

### Study Population

The study was conducted in Dong Thap province (Mekong Delta of Vietnam), from December 2017 to March 2018. The Mekong Delta is a relatively homogeneous agro-ecological region, and Dong Thap province is representative of this region. We chose the five (of 12) districts with the highest poultry populations, and focused on the three main types of poultry production in this area. The production cycle was typically 4 months for meat chickens, 2–3 months for meat ducks, and 2–3 years for free-ranging ducks. The study population consisted of (a) farmers, including owners of chicken, meat duck and free-grazing duck flocks, and (b) farmer' advisors, comprising veterinary drug shop owners, CAHWs and government veterinarian.

**Figure 1:**
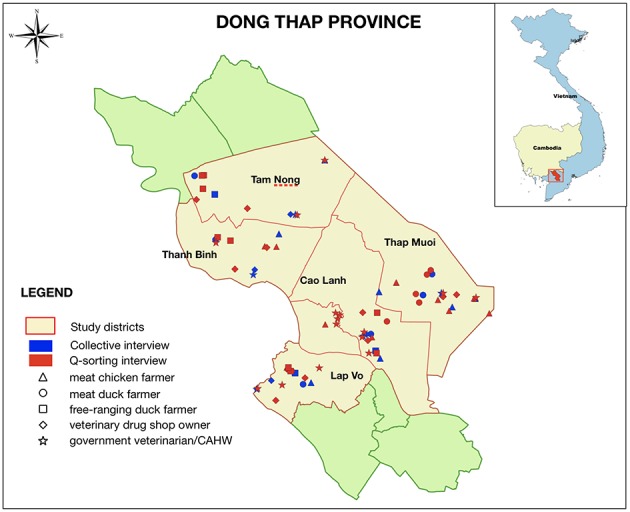
Map of study areas in Dong Thap province. Blue: geographical location of CIs; red: geographical location of participants of Q-sorting interviews; triangle: chicken farmers; circle: meat duck farmers; square: free-grazing duck farmers; rhombus: veterinary drug shop owners; star: government veterinarian/CAHWs.

Farmers and veterinary drug shop owners were randomly selected from the official census held at the sub-Department of Animal Health and Production in Dong Thap (SDAH-DT). Government veterinarian/CAHWs were also randomly selected from the staff list. We aimed to select 250 participants of the five types of stakeholders (50 per district), organized into 25 semi-structured collective interviews (CIs) (five per district). The term “CI” was chosen over “FGI” (focus group interview), since the group of participants was heterogeneous and we were more seeking for a consensus in the answers, rather than exploring controversial points of view. The latter is normally applicable to FGI. Each CI session included 10 participants of one type of stakeholder. The number of CI chosen for each type of stakeholder (five) was based on (a) the concept of “saturation point,” that estimates that 90% themes within a research topic are normally discoverable by conducting three to six group interviews with each type of stakeholder (20); and (b) the objective of capturing the diversity opinion of farmers who raised different types of poultry and lived in different districts within the province. For Q-sorting, 55 participants of CIs were randomly selected and were invited to participate in the Q-sorting phase by conducting individual interviews. This number of participants was based on the sampling criteria described by Brown (14). The selection of participants formed a heterogeneous group based on type of production, gender, age, education level, location and experience in raising poultry (farmers). In addition, five government veterinarians were invited to take part in the Q-sorting step, since they are thought to play a very important role in Vietnamese animal production. All five interviewers and facilitators had previously been trained in PE and Q-sorting methodologies. All steps were conducted in Vietnamese since over 95% of the population in this province are ethnic Vietnamese. The interview sessions (CI or Q-sorting interview) took about 1 h each. Data were collected during the discussions with a digital voice recorder, and during the PE exercises information was recorded using written notes and pictures. All participants were initially contacted by staff affiliated to the SDAH-DT. For each interview (CI or Q-sorting interview), written informed consent was obtained from all participants before enrolment. The location of the interview sessions is shown in Figure 1.

The study was performed in several sequential steps (Supplementary Figure 1) (17).

### Collection of Descriptive Data Using PE Tools

Qualitative and semi-quantitative data about existing opinions about AMU were collected during CI with farmers and their advisors using both open-ended questions and a checklist organized in four thematic areas (Supplementary 1): (a) Characterize diseases in poultry farms (chicken and duck farmers separately), and describe farmers' strategies to prevent and control them; (b) Identify the timing of AMU (chicken and duck farmers separately); (c) Identify sources of advice and procurement of antimicrobials to farmers (farmers and their advisors); and (d) Identify positive and negative opinions on AMU (farmers and their advisors). Various participatory tools were used to collect the data. These included pair-wise ranking for (a), time line tool for (b), proportional piling for (b) and (c), and flow-chart for (d). The PE data collection was performed following published guidelines (21). At the end of the discussions on each thematic area, a consensus was sought. The facilitators summarized the main CI findings and asked: “*Do you all agree, or would you like to change something*?” Minority opinions were discussed in all cases, after which the group was asked to accept or reject those opinions.

### Q-Sorting Interviews and In-Depth Post Q-Sorting Interviews

The raw data gathered in the PE phase were used to generate a list of statements. This process included: screening, summarizing the data, creating statements, and modifying statements in line with research team's opinions. The Q-sorting interview process has been described by Truong et al. (17). In short, participants were invited to read, score, and allocate statements into a quasi-normal grid of 46 boxes according to their option. Statements were scored from −3 (extremely disagree) to +3 (extremely agree) (i.e., seven discrete options). After Q-sorting interview, additional questions were asked to participants to clarify the reasons behind their choice of extreme values for statements.

### Statistical Analyses

The non-standardized data (semi-quantitative) collected from the pair-wise ranking exercise were transformed and standardized with a rank-score process (21). CI participants were asked to list poultry diseases important in their area and rank them according to their importance. This rank was transformed into a score, and results were averaged across CI for each disease using the median and inter-quartile range. CI participants were also asked whether they would use antimicrobials should they encounter each of the diseases listed. The information generated was converted into a probability of AMU conditional to each disease listed being present, and binomial confidence intervals were calculated around these estimates. Other descriptive (semi-quantitative) data were summarized using median score (MS), interquartile range (for proportional piling exercise), and percentage (for frequency of information from the flow-chart exercise) where appropriate. Data from each Q-sorting interview were introduced into two correlation matrices (one for farmers and one for farmers' advisor group) that included statements as observations and participants as variables (22, 23) (Supplementary Figure 2). Principal component analysis (PCA) was performed on these correlation matrices in order to shortlist a number of factors (3–10) for the next step of analysis (17, 24). The number of factors selected was based on the level of heterogeneity of participants' views, subjectively evaluated by the researchers (25). The correlation matrices generated were subjected to factor analysis separately in order to identify discourses that best characterized clusters of participants (25, 26) as described by Truong et al. (17). Rotation of *k* factors (chosen from 3 to 10) was carried out during the factor analysis on the basis of (a) the best factor combination could explain 40% of cumulative percentage of variation; and (b) each factor comprised at least 5% of the total Q sort that loaded distinctly and significantly (14, 17, 27). Respondents who were assigned to more than one factor were considered as confounders. The respective score of each statement were recalculated through factor analysis process and it represented the relative score of one statement given by one particular discourse. The outcome was *k* discourses which were represented by *k* selected factors at the beginning. These discourses were a hypothetical Q-sorting that had been reconstructed from the factor scores (17, 25) (Supplementary 2, Supplementary Table 2). Statements were regarded as consensus points when the difference between the scores attained in any pair of factors were not statistically significant (based on the standard error of differences) (27). Transcripts from CIs and Q-sorting interviews were stored and extracted using the “rqda” package in R (28). Those data were not being analyzed statistically but were integrated in discussion section as explanation for the results obtained from exercise in the field. All data analyses were performed using R statistical software (29).

## Results

### Study Population

A total 26 CIs with 198 participants were conducted: five CIs with veterinary drug shop owners (34 participants), five CIs with government veterinarians/CAHWs (39 participants), seven CIs with chicken farmers (49 participants), six CIs with meat duck farmers (30 participants), and three CIs with free-ranging duck farmers (46 participants). The actual number of CIs and participants were slightly different from the planned number due to unpredictable field constrains. Of the 60 participants that had been invited in the Q-sorting interview, six were removed from the analysis either because of their misunderstanding of the Q-sorting instructions or unwillingness to complete the procedure. The analysis therefore included 28 farmers and 26 advisors. The demographic features of participants are shown in Table 1.

**Table 1:** Demographic description of participants involved in CI and Q-sorting interviews phases of the study.

	**Collective interviews participants**					**Q-sorting interviews participants**				
	**Total (*n* = 198)**	**Chicken farmers (*n* = 49)**	**Meat duck farmers (*n* = 30)**	**Free-ranging ducks farmers (*n* = 46)**	**Farmers' advisors (*n* = 73)**	**Total (*n* = 54)**	**Chicken farmers (*n* = 11)**	**Meat duck farmers (*n* = 8)**	**Free-ranging duck farmers (*n* = 9)**	**Farmers' advisors (*n* = 26)**
Age in years [median [interquartile range]]	41.0 [34.0–50.0]	45.0 [35.0–54.0]	44.0 [37.0–51.5]	42.5 [37.0–47.8]	35.0 [33.0–43.0]	43.0 [34.3–51.0]	51.0 [48.5–62.0]	41.0 [32.0–51.8]	46.0 [42.0–53.0]	38.0 [33.5–43.0]
**GENDER**										
Male (%)	178 (89.9)	43 (87.8)	29 (96.7)	45 (97.8)	61 (83.6)	48 (88.9)	10 (90.9)	8 (100.0)	9 (100.0)	21 (80.8)
Female (%)	20 (10.1)	6 (12.2)	1 (3.3)	1 (2.2)	12 (16.4)	6 (11.1)	1 (9.1)	0 (0.0)	0 (0.0)	5 (19.2)
**DISTRICT**										
Cao Lanh (%)	43 (21.7)	10 (20.4)	7 (23.3)	9 (19.6)	17 (23.3)	15 (27.8)	2 (18.2)	2 (25.0)	2 (22.2)	9 (34.6)
Lap Vo (%)	43 (21.7)	7 (14.3)	9 (30.0)	11 (23.9)	16 (21.9)	10 (18.5)	1 (9.1)	2 (25.0	2 (22.2)	5 (19.2)
Tam Nong (%)	40 (20.2)	9 (18.4)	0 (0.0)	18 (39.1)	13 (17.8)	7 (13.0)	0 (0.0)	0 (0.0)	3 (33.3)	4 (15.4)
Thanh Binh (%)	25 (12.6)	5 (10.2)	0 (0.0)	8 (17.4)	12 (16.4)	8 (14.8)	2 (18.2)	0 (0.0)	2 (22.2)	4 (15.4)
Thap Muoi (%)	47 (23.7)	18 (36.7)	14 (46.7)	0 (0.0)	15 (20.5)	14 (25.9)	6 (54.5)	4 (50.0)	0 (0.0)	4 (15.4)

### Descriptive Data

**Figure 2:**
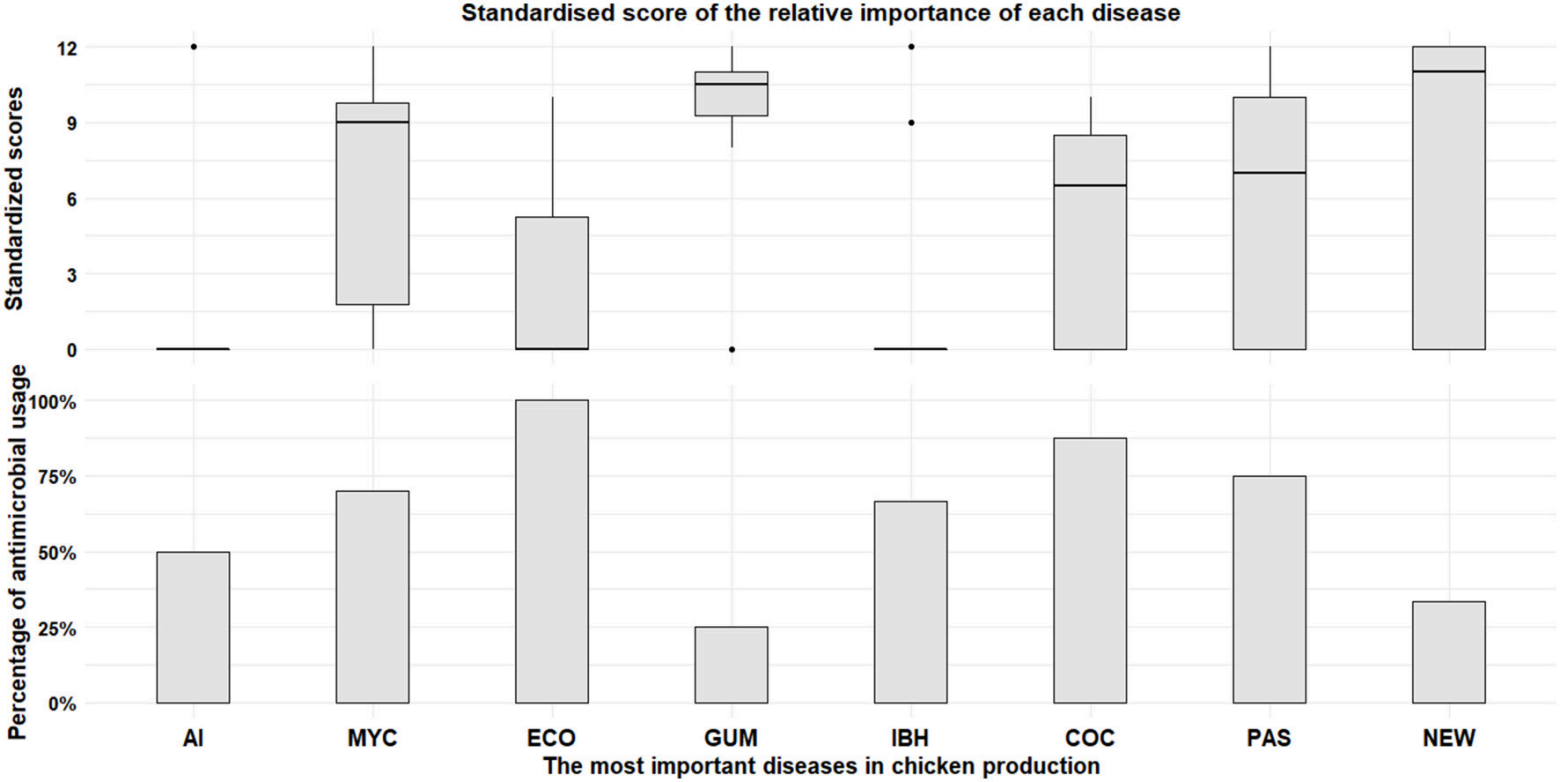
Classification of the most important diseases (top) and frequency of AMU conditional to disease present in their farm (bottom) for chicken farms. AI, Highly Pathogenic Avian Influenza; MYC, Mycoplasmosis; ECO, *Escherichia coli*; GUM, Gumboro; IBH, Inclusion Body Hepatitis; COC, Coccidiosis; PAS, Pasteurellosis; NEW, Newcastle.

**Figure 3:**
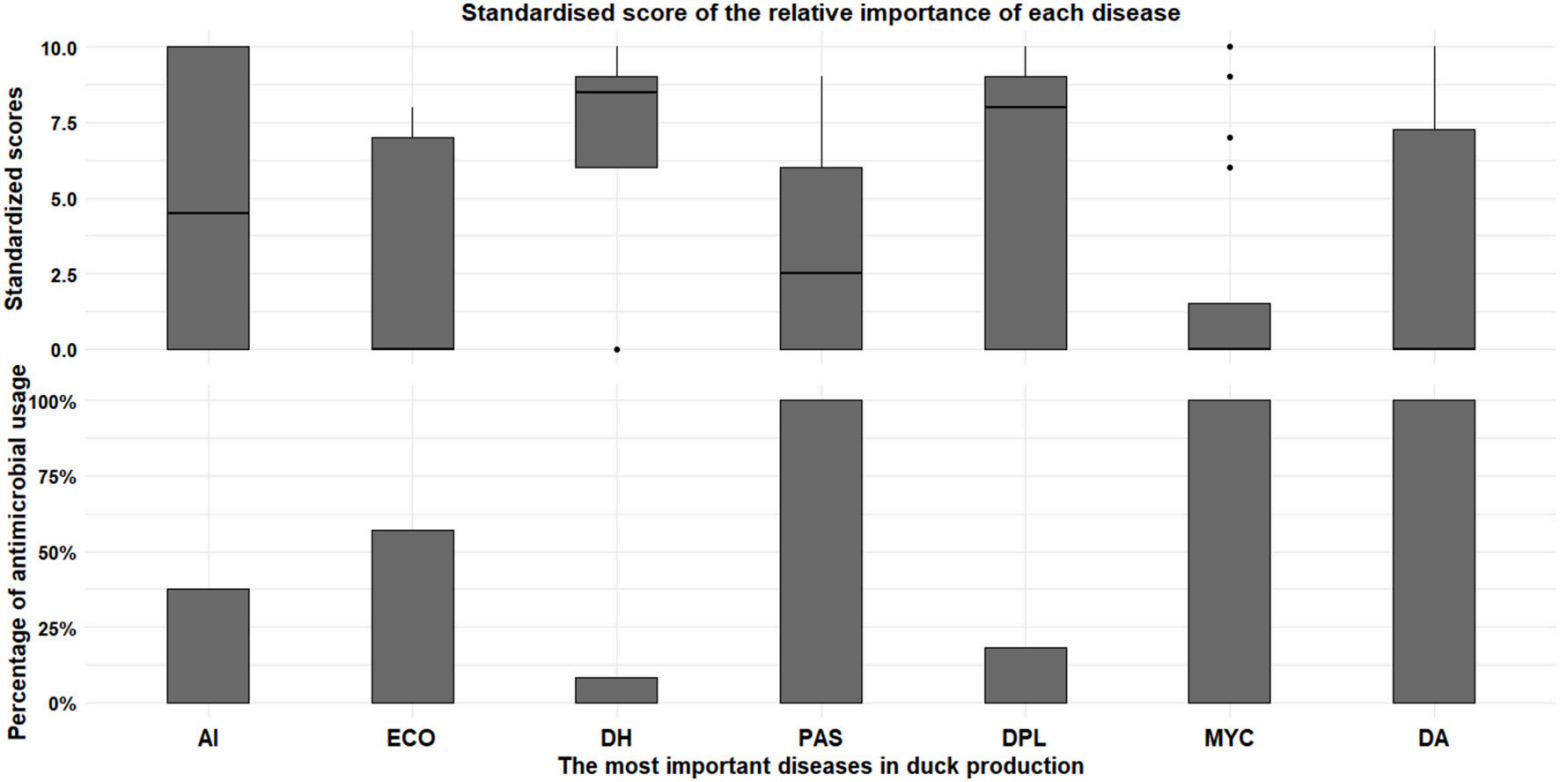
Classification of the most important diseases (top) and frequency of AMU conditional to disease present in their farm (bottom) for duck farms. AI, Highly Pathogenic Avian Influenza; ECO, *Escherichia coli;* DH, Duck Hepatitis; PAS, Pasteurellosis; DPL, Duck Plague; MYC, Mycoplasmosis; DA, Duck Aspergillosis.

The CIs identified a total of 15 poultry infectious diseases (data not shown). Diseases were described using their local names (often designing the etiological agent). The three chicken diseases that ranked highest across all CIs were: Gumboro disease; mycoplasmosis; and Newcastle Disease (Figure 2). The duck diseases that ranked highest were duck hepatitis and duck plague (Figure 3). The CIs indicated that antimicrobial use if flocks were affected by bacterial disease was greatest for pasteurellosis (87.5%; i.e., 14 CIs would use antimicrobials among 16 CIs reporting this disease), colibacillosis (72.7%; 8/11), and mycoplasmosis (78.6%; 11/14). For viral diseases usage was related to: Highly Pathogenic Avian Influenza (HPAI) (40.0%; 4/10); ND (33.3%; 3/9); Gumboro disease (25.0%; 3/12); Duck plague (18.2%; 2/11); Duck hepatitis (8.3%; 1/12). Other causative agents and usage included hepatitis (66.7%; 2/3), coccidiosis (87.5%; 7/8), and aspergillosis (100.0%; 6/6). A total of 15.0% of CIs reported prophylactic antimicrobial use during seasonal transitions.

**Figure 4:**
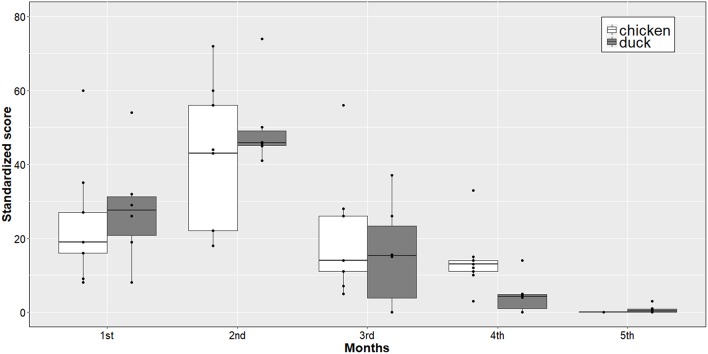
Stakeholders' opinion about source of advice to farmers on AMU. White: Farmers' source of advice (farmers' opinion); Gray: Farmers' source of advice (Farmers' advisors opinion); VDSO, Veterinary drug shops owners; G.V/CAHW, Government Veterinary/Community Animal Health Workers; SPPC, Salespersons of pharmaceutical companies; SPFC, Salespersons of feed companies; FC, Farmer colleagues; FKE, Own farmer' knowledge/experience.

In quantitative terms, most of the antimicrobials were administered during the second month of the production cycle (MS 43.0 and 45.5% for chicken and duck production, respectively), followed by the first month of the production cycle (MS 19.0 and 29.0%) (Figure 4).

**Figure 5:**
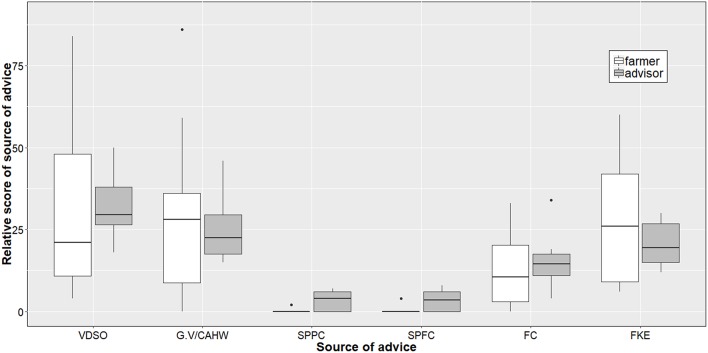
Timing of AMU during production cycle.

The most trusted sources of advice to farmers were government veterinarian/CAHWs (MS = 28.0), their own knowledge/experience (MS = 26.0), the veterinary drug shop owners (MS = 21.0), and sales persons from pharmaceutical and feed companies (MS = 0.0). The farmers' advisor group ranked the veterinary drug shop owner as the most important source of advice to farmers (MS = 29.5), followed by government veterinarians/CAHWs (MS = 22.5), the farmers' own knowledge/experience (MS = 19.5), sales persons of pharmaceutical companies (MS = 4.0) and sales persons of feed companies (MS = 3.5) (Figure 5). Five positive and seven negative outcomes of AMU were identified. Similarly, eight positive and five negative outcomes were identified because of not using antimicrobials, respectively (Table 2).

**Table 2:** Opinions about positive and negative aspects with regards to AMU reported in farmers' CI (16) and in farmers' advisors' CI (10).

	**Farmers' (CI** **=** **16) opinions**				**Farmers' advisors' (CI** **=** **10) opinions**			
	**Positive aspects**	**%**	**Negative aspects**	**%**	**Positive aspects**	**%**	**Negative aspects**	**%**
Using antimicrobial	Disease treatment	100	Increases production costs	68.8	Disease prevention	80.0	Treatment failure	70.0
	Disease prevention	87.5	Reduces productivity	62.5	Avoids mortality	80.0	Reduces productivity	50.0
	Reduces mortality	50.0	Increases feed costs	50.0	Disease treatment	70.0	Antimicrobials residues in meat and egg	40.0
	Keeps flocks healthy	37.5	Treatment failure	37.5	Increases income	30.0	Increases production costs	40.0
	Increases income	6.3	Increases labor costs	25.0			Increases feed costs	40.0
			Risk of using counterfeit drugs	18.8				
			Antimicrobials residues in meat and egg	18.8				
Not using antimicrobial	Saves money through decreases costs of production	50.0	Increases mortality due to disease	62.5	Saves money through decreases costs of production	50.0	Increases mortality due to disease	60.0
	Increases productivity	31.3	Weakens the immune system	6.3	Increases meat and egg quality	40.0	Reduces productivity	10.0
	Provides safe products	31.2			Flock grows faster	20.0	Reduces income of vet drug-shop owners	10.0
	More time for other activities	18.8			No antimicrobials residues in meat and egg	10.0	Unable to cure diseases	10.0

### Q-Sorting Interviews

Based on the list of opinions from different stakeholders, 46 final statements were generated, representing the spectrum of opinions on AMU around four thematic areas: (a) Farmers' confidence in antimicrobials as a tool for prevention, treatment or growth promotion; (b) Antimicrobial administration logistics; (c) Costs of the antimicrobials used; and (d) Impact of AMU/AMR on animal health/productivity and human health (See list of the statements related to each of these areas in Supplementary Table 1).

### PCA and Factor Analysis

**Figure 6:**
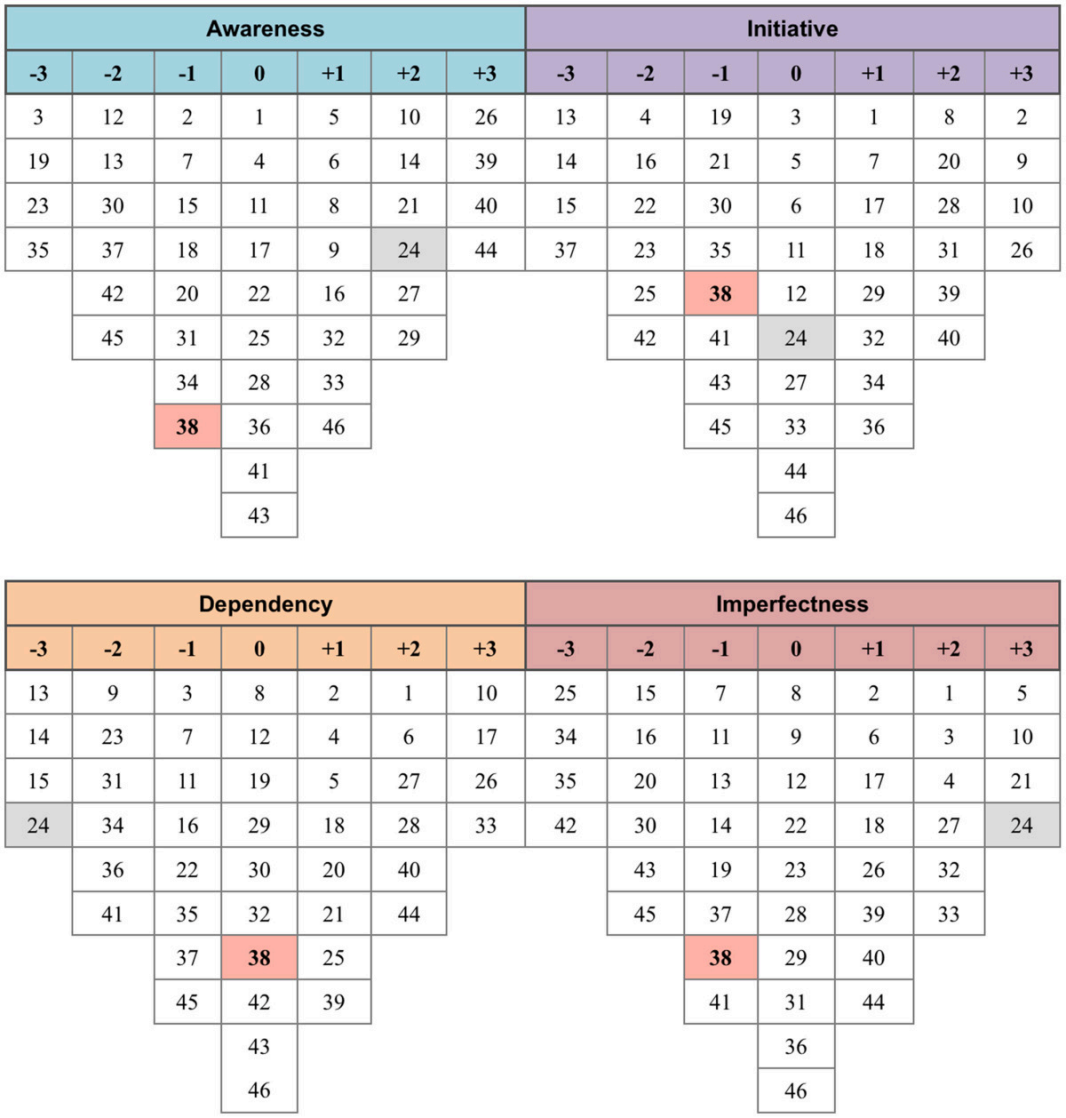
Four discourses summarizing farmers' opinions. Number from +3 to −3: Score in one discourse; Number in red cells: Consensus statements; Number in gray cells: Distinguished statements (see Supplementary Table 1 for detailed information).

**Figure 7:**
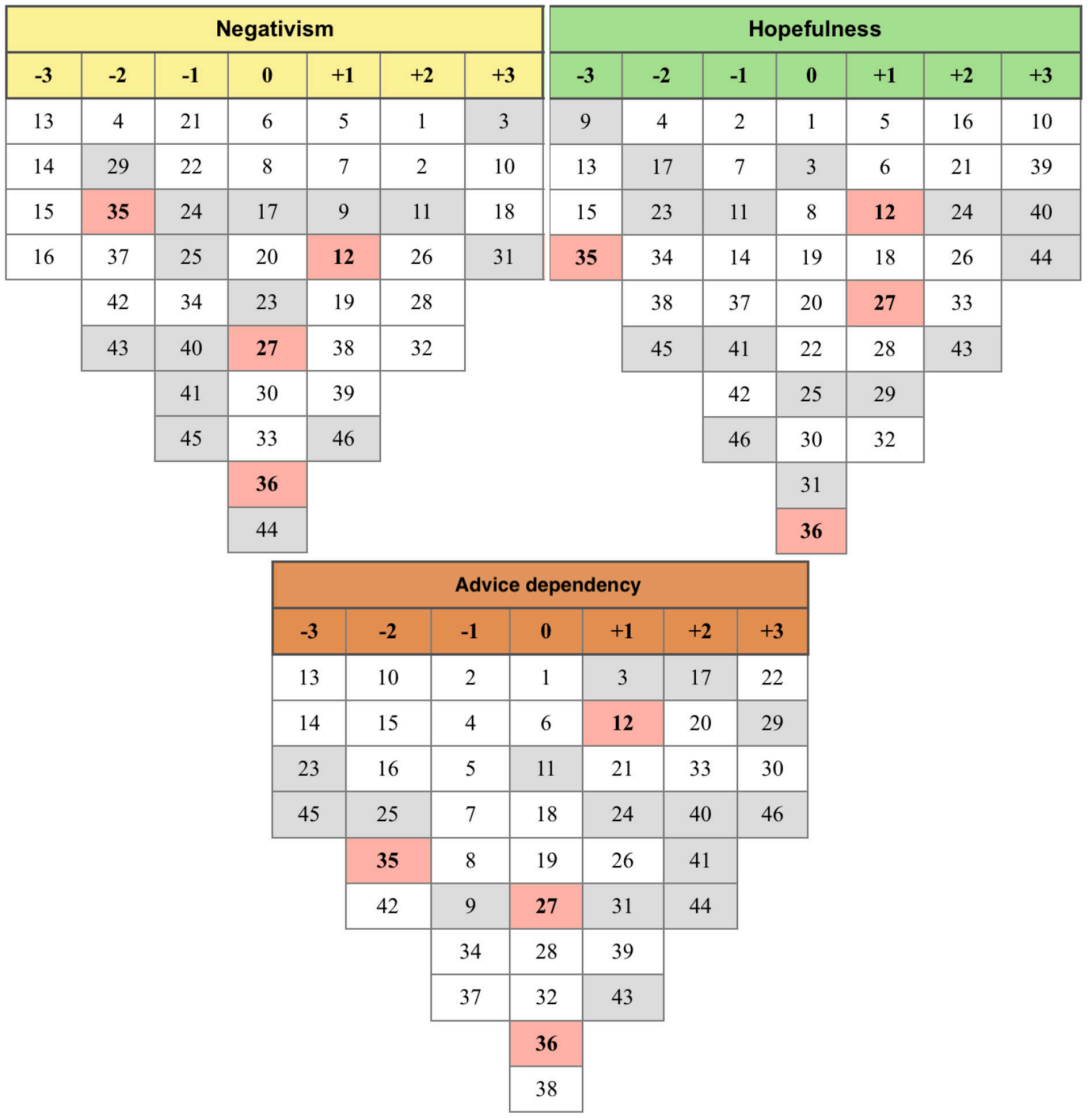
Three discourses summarizing farmers' advisors' opinions. Number from +3 to −3: Score in one discourse; Number in red cells: Consensus statements; Number in gray cells: Distinguished statements (see Supplementary Table 1 for detailed information).

Among the farmer group, four discourses (F1–F4) were identified. These explained 17, 15, 13, and 10% of the total variability (55% cumulative variance). Among farmers' advisors, three discourses (A1–A3) were identified, explaining 18, 17, and 15% of the total variability (50% cumulative variance). Six respondents were considered as confounders. The discourses were labeled based on the score attained on some relevant statements. The statement numbers followed by their respective scores are shown within brackets (i.e., 46, −2 means statement number is 46 and its score is −2). The summary of the reconstructed Q-sorting from a total of seven discourses in both groups was shown in Figures 6, 7.

### Discourse Description

Discourse F1 represented farmers who displayed knowledge of AMU in poultry production (“Awareness”). Farmers allocated to discourse F2 were reliant on antimicrobials to raise poultry (“Dependency”). Discourse F3 represented farmers who freely use antimicrobials without consulting anyone else (“Initiative”). Discourse F4 constituted a group of farmers who had limited knowledge on AMU (“Imperfectness”). Advisors following discourse A1 (“Negativism”) thought that farmers generally lack knowledge on AMU. Discourse A2 (“Hopefulness”) was assigned to advisors who believed that farmers' knowledge and attitude although inadequate, will eventually improve. Finally, discourse A3 (“Advice dependency”) characterized advisors that claimed that farmers were dependent on external advice.

### Farmers' Discourse F1: “Awareness”

Eight of 26 farmer respondents (30.8%) contributed to the “Awareness” discourse, which included four chicken farmers, three duck farmers, and one free-grazing duck farmer. They reported that they never used antimicrobials as the first choice of treatment if they did not know the reason why their birds were sick (3, −3), and reported that they never used antimicrobials for disease prevention (14, +2). These respondents knew that overuse of antimicrobials leads to their loss of effectiveness (44, +3). They believed that improper AMU might cause sudden death in some cases (40, +3). Furthermore, these farmers appreciated the importance of biosecurity, and completely disagreed with the notion that when flocks are given antimicrobials, there was no further need for other disease control methods (23, −3; 24, +2). They trusted the advice of government veterinarians/CAHWs about AMU (21, +2), and they did not seek advice on AMU from neighboring farmers (19, −3). Some also believed that using antimicrobials to treat disease by themselves was more costly than seeking veterinary advice (30, −2).

### Farmers' Discourse F2: “Dependency”

Seven farmers (26.9%) (three chicken farmers, one meat duck farmer, and three free-grazing duck farmers) contributed to the “Dependency” discourse. Antimicrobials were always used by these farmers both for prevention and treatment of disease (13, −3; 14, −3; 15, −3). Even if flocks were kept in conditions of high biosecurity, they would still use antimicrobials for prevention (24, −3), since AMU gave them a sense of security (1, +2). They also reported that they medicated their bird as soon as they heard of a disease outbreak spreading in their area (17, +3). They also perceived that the costs of antimicrobials were too high relative to overall total production costs (36, −2). However, when asked about how a potential three- to four-fold increase in price would affect their AMU practices, these farmers stated that they would not change their current AMU practice (41, −2). They trusted the advice on AMU from government veterinarians/CAHWs (21, +1). They believed that antimicrobials offered by veterinarians were of good quality (6, +2), and were more willing to allow veterinarians treat their sick flocks rather than undertaking this task by themselves (31, −2).

### Farmer's Discourse F3: “Initiative”

The “Initiative” discourse corresponded to six respondents (23.1%) owning larger flocks (two chicken farmers, one meat duck, and three free-grazing duck farmers with more than 500 heads each). Farmers assigned to this discourse frequently relied on antimicrobials for prevention and treatment of disease in their flocks (13, −3; 14, −3). They felt secure when their flocks were given antimicrobials for prevention (2, +3). They reported that in spite of good farming practices, antimicrobials were still necessary as prophylactic and therapeutic agents (26, +3). They relied on their own knowledge and experience in terms of choosing the most appropriate antimicrobial product to treat their flocks (8, +2; 31, +2). They expressed a lack of trust in private veterinary, veterinary drug shop owners (22, −2).

### Farmer's Discourse F4: “Imperfectness”

The “Imperfectness” discourse was assigned to five respondents (19.2%) (two chicken farmers, two meat duck farmers, and one free-grazing duck farmer) who generally trusted the advice of government veterinarians/CAHWs (21, +3). They believed that antimicrobials were not needed if birds were raised in conditions of good biosecurity (24, +3). They followed the recommended full treatment course indicated in the product label (25, −3). They believed that preventing disease by vaccination would be more cost-effective than medicated sick birds (42, −3). However, if their flocks became sick they applied antimicrobials to the whole flock (including healthy-looking birds) as their first choice (3, +2; 16, −2). They expressed relieve after their sick flock became treated with antimicrobials (1, +2).

### Advisors' Discourse A1: “Negativism”

Eight respondents in this group (36%) (two veterinary drug shop owners, six government veterinarians/CAHWs) believed that farmers continued to have considerable misunderstandings about AMU. They assumed that farmers resorted to use antimicrobials as their first choice when dealing with disease problems (3, +3), farmers made their decisions about AMU without any input from external advisors (18, +3; 31, +3). AMU gave farmers a sense of security, reducing their stress and increasing their confidence in their production system (1, +2; 2, +2). Most farmers used antimicrobials for disease treatment and prevention, as well as for growth promotion (13, −3; 14, −3; 15, −3). They considered that farmers never followed the recommended dosing instructions in the label when treating animals (11, +2). When a part of flock was sick, instead of placing segregating it (i.e., in a sick pen), farmers would rather give antimicrobials to the whole flock in an attempt to prevent disease spreading (16, −3).

### Advisors' Discourse A2: “Hopefulness”

The advisors' “Hopefulness” discourse described the actions and beliefs of eight respondents (36%) (five veterinary drug shop owners, three government veterinarians/CAHWs). Respondents in this discourse believed that farmers would not use antimicrobials for prevention if good biosecurity were applied in their poultry farming (24, +2; 17, −2). These advisors believed that farmers knew that overuse and misuse of antimicrobials resulting in their loss of effectiveness (i.e., AMR) (44, +3), contributing to decreasing animal productivity (39, +3). They believed that farmers mostly trust in the advice from government veterinarian/CAHWs (21, +2).

### Advisors' Discourse A3: “Advice dependency”

The “Advice dependency” discourse was attributed to six advisors (23.1%) who claimed that farmers generally had poor knowledge of good AMU practices, and were dependant on advice provided by private veterinarians and veterinary drug shop owners (22, +3). This group believed that farmers used antimicrobials to both prevent and treat disease in their flocks (13, −3; 14, −3). They believed that farmers would use antimicrobials over the entire production cycle even if only a proportion of birds in the flocks were sick (16, −2), and would also use them when hearing of a disease outbreak in the surrounding area (17, +2). They also thought that farmers would decrease AMU when the price of antimicrobials increased (41, +2).

### Consensus Points From Q-Sorting Analysis

Among farmers, only one consensus point was found: they all believed that the cost of antimicrobials was more expensive than the cost of other biosecurity methods as currently applied in their farms (Statement 38). All advisors agreed on the following statements: (a) The cost of treating flocks using antimicrobials was more expensive than the cost of using vaccines for prevention (Statement 35); (b) Farmers would use antimicrobials as first choice when dealing with their sick flock over other practices such as segregating sick birds and early mortality culling (Statement 12); (c) They all had no opinion about the price of antimicrobials in relation to the total production costs (Statement 36); and (d) They had no knowledge about the timing when antimicrobials are most commonly used by farmers (Statement 27).

## Discussion

Through the Q-sorting we identified four distinct attitudes (discourses) on AMU among farmers. They were all relatively evenly distributed, with each of these accounting for 19.2–30.8% of farmers investigated. The “Awareness” discourse was the most prevalent (30.8%). Farmers assigned to this discourse were aware of the limitations and issues regarding AMU/AMR, and reported never using antimicrobials to prevent disease. On the other extreme, a group of farmers (26.9%) were assigned to a discourse (“Dependency”) that reflected total dependency on antimicrobial for raising their flocks.

In contrast with traditional questionnaire-based interviewing methods, PE allows farmers to freely explore the topics by themselves, and therefore it was considered to be most appropriate in this setting. This study fulfilled the criteria outlined for PE studies described by Catley et al. (12): (1) active involvement of respondents allowing them to express their opinions and perceptions; (2) local knowledge about concrete problems collected from CI was used to generate statements in Q-sorting interviews (bottom-up approach); (3) the collected data was triangulated during interviews and between interviews with the help of open-ended questions. However, the choice of specific themes proposed in the CI phase was naturally influenced by the experience and knowledge of the researchers on AMU/AMR. Farmers were selected from a farm census maintained and updated annually by SDAH-DT. The census is not balanced with regards to gender, since the overwhelming majority of registered farm owners were male. The reason is that in rural Vietnamese households, the named farm owner is typically the husband, even if the responsibility for tending poultry flocks often lies within the wife. During the initial telephone call to the owner as part of the recruitment process, we aimed to achieve a more balanced sample by inviting the person (male or female) directly responsible for tending the poultry flocks. In spite of that, 90% of the participating farmers were male. We believe this is due to the fact that in Vietnamese culture it is normally the male who liaises with external agents. Because of this, we might not have captured all of the women's opinions related to the study research questions. Another potential bias might be an under-representation in our sample of part-time farmers or farmers having other occupations in addition to tendering poultry. We used the same 46 statements to investigate attitudes on AMU in both farmers and their advisors. Since some farmers' advisors had limited knowledge/experience on AMU, this might have been a source of bias in the Q-sorting interviews. Therefore, some of their opinions about farmers' attitudes may be more a reflection of circulating views than actual hands-on advisory experience.

Farmers reported that the highest amounts of antimicrobials were used during the first half of the production cycle. This corresponds to the brooding and early grow-out periods. This period often involves changes in housing and feeding conditions and is perceived to be the period when flocks are at their highest risk of disease. Increased risk of disease was also reported during at two critical time points: during vaccination against viral diseases to control secondary bacterial infections, and during transition from the dry to the rainy season. Farmers believed that using antimicrobials during this period helped them reduce their anxiety about the risk of diseases. Keeping the flock healthy and maximizing number of live birds sold to the market (outputs) whilst lowering the input costs as much as possible are the two main targets of poultry farmers (30). Therefore, in the eyes of many farmers, antimicrobials are seen as part of “good farming practice.” Because of this, they might also neglect other disease control measures (31).

All farmer participants agreed that the costs incurred in AMU were higher than the cost of biosecurity and disease control methods as were currently implemented in their flocks (Statement 38). They however recognized that should they upgrade biosecurity and other disease control methods, this would eventually lead to greater reductions in the incidence of disease and therefore this would result in reduced need of antimicrobials. From our observations, biosecurity methods implemented by most farmers in the area typically consist of keeping pens visibly clean and regularly applying disinfectants. Most farmers are also regularly supplied with disinfectants and HPAI vaccines free of charge by the veterinary authorities. Farmers often think that disease control programmes supported by the veterinary authorities are crucial in reducing the risks to their flock. A recent study showed that Vietnamese cattle farmers felt more secure after taking part in an official vaccination campaign (17). However, it has been shown that even well-established vaccination campaigns such as HPAI in poultry may in fact provide limited protection against circulating viruses (32). Therefore, the provision of vaccines by the veterinary authorities may have a negative impact by creating a false sense of security.

The fact that some farmers in the area were prepared to accept a three- to four-fold increase in the price of antimicrobials suggests that there is a potential for revising pricing policies, increasing them to deter AMU in situations when antimicrobials are not strictly necessary. However, rapid increases in prices could potentially result in the creation of a black market for antimicrobials.

Compared with private veterinarians and veterinary drug shop owners, government veterinarians/CAHWs were ranked by famers as a more trustworthy source of information on AMU (Figure 3). Farmers also reported that they had more regular contact with private veterinary drug shop owners than with other stakeholders. In each commune in the area, there is typically only one or two government veterinarian/CAHW, compared with three-six veterinary drug shop owners. Veterinary drug shop owners have a vested interest in antimicrobial sales. Many smallholder farmers tend to rely on their own experience with regards to AMU (“Initiative” discourse). Participants in this discourse would just ask for advice when they encounter more serious disease. This attitude was closely linked to large (>500 heads) free-grazing (mobile) duck flocks that typically travel long distances to graze on paddy fields. Farmers of these flocks feel they need to be prepared to administer antimicrobials should their flocks become diseased in locations far from their “local” veterinary drug shops.

The lack of understanding of animal health advisors on poultry farm-level economics is of concern. The advisors' belief that poultry production costs could be easily reduced by adopting alternative disease control practices contrasts with the farmers' understanding of the costs of AMU vs. biosecurity and vaccination. This lack of agreement is an area for education and training policy, whilst requiring further research on the economics of AMU and alternative animal health interventions.

## Conclusions and Recommendations

A combination of PE and Q-sorting approach provided meaningful insights into the attitudes of the different stakeholders involved in the procurement and usage of antimicrobials in poultry farming. Through the study of 203 participants in Dong Thap province, we characterized the purpose of AMU (treatment and prevention), the timing associated with higher AMU levels (first half of the production cycle), and the cost of AMU (cheaper than other biosecurity methods). Farmers were aware of good husbandry practices (including good biosecurity) as effective disease control tools. However, these practices were regarded as expensive alternatives to AMU, and their implementation would require sustained training efforts. Given that farmers have relatively greater trust of official government veterinarians, we recommend reinforcing their advisory role in order to counteract the influence of veterinary drug shop owners (currently the first point of contact to farmers). From an educational perspective, veterinarians, and animal health advisors need guidance on farm-level economics of poultry farming and a better understanding of costs. This reinforced advisory capacity should focus on improving overall farming practices whilst discouraging prophylactic AMU. Given the complexity and diversity of poultry production systems in the Mekong Delta region of Vietnam, we recommend scaling up research on socio-economic factors driving AMU in small-scale farms in the region. Furthermore, the clear gender imbalance evidenced in our study population suggests that more research is needed to understand the perceptions and views of female Vietnamese farmers with regards to AMU.

## Data Availability

All datasets generated for this study are included in the manuscript and/or the Supplementary Files.

## Ethics Statement

The ViParc project has been granted ethics approval by the Oxford Tropical Research Ethics Committee (OXTREC) (Ref. 5121/16).

## Author Contributions

HD, DT, FG, JC-M, and JR designed the study, contributed to the analyses, and drafted the manuscript. VD, VN, and TB designed the data collection instrument and drafted the manuscript. CR aided in reviewing results and provided discussion comments. AB and GT reviewed the results and drafted the manuscript. The manuscript has been read and approved by all authors.

### Conflict of Interest Statement

The authors declare that the research was conducted in the absence of any commercial or financial relationships that could be construed as a potential conflict of interest.

## Supplementary Material

Supplementary 1 Checklist used for CI-participatory epidemiology approach.

Supplementary 2 Script for Q-sorting interviews analysis run in R program.

Supplementary Table 1 List of statements used in this study and their score according to each factor/discourse after Q-sorting analysis.

Supplementary Table 2 Raw data of Q-sorting interviews.

Supplementary Figure 1 Flow chart of study steps.

Supplementary Figure 2 Flow chart of statistical analyses steps.

## Abbreviations

AMU: Antimicrobial usage
PE: Participatory epidemiology
PCA: Principal Component Analysis.

## References

[1] O'Neill J Antimicrobials in Agriculture and the Environment - Reducing Unnecessary Use and Waste. (2015). Available online at: https://amr-review.org/sites/default/files/Antimicrobials%20in%20agriculture%20and%20the%20environment%20-%20Reducing%20unnecessary%20use%20and%20waste.pdf

[2] Cuong N Padungtod P Thwaites G Carrique-Mas J. Antimicrobial usage in animal production: a review of the literature with a focus on low- and middle-income countries. Antibiotics. (2018) 7:75. 10.3390/antibiotics7030075 30111750 PMC6164101

[3] Kumar V Gupta J. Prevailing practices in the use of antibiotics by dairy farmers in Eastern Haryana region of India. Vet World. (2018) 11:274–80. 10.14202/vetworld.2018.274-280 29657416 PMC5891839

[4] Van Boeckel TP Brower C Gilbert M Grenfell BT Levin SA Robinson TP. Global trends in antimicrobial use in food animals. Proc Natl Acad Sci USA. (2015) 112:5649–54. 10.1073/pnas.1503141112 25792457 PMC4426470

[5] Carrique-Mas J Van NTB Cuong NV Truong BD Kiet BT Thanh PTH. Mortality, disease and associated antimicrobial use in commercial small-scale chicken flocks in the Mekong Delta of Vietnam. Prev Vet Med. (2019) 165:15–22. 10.1016/j.prevetmed.2019.02.005 30851923 PMC6418316

[6] Carrique-Mas JJ Trung NV Hoa NT Mai HH Thanh TH Campbell JI. Antimicrobial usage in chicken production in the Mekong Delta of Vietnam. Zoonoses Public Health. (2015) 62:70–8. 10.1111/zph.12165 25430661

[7] Nguyen NT Nguyen HM Nguyen CV Nguyen TV Nguyen MT Thai HQ. Use of colistin and other critical antimicrobials on pig and chicken farms in Southern Vietnam and its association with resistance in commensal *Escherichia coli* bacteria. Appl Environ Microbiol. (2016) 82:3727–35. 10.1128/AEM.00337-16 27084016 PMC4907207

[8] Ström G Boqvist S Albihn A Fernström L-L Andersson Djurfeldt A Sokerya S. Antimicrobials in small-scale urban pig farming in a lower middle-income country – arbitrary use and high resistance levels. Antimicrob Resist Infect Control. (2018) 7:35. 10.1186/s13756-018-0328-y 29541447 PMC5842516

[9] Ström G Halje M Karlsson D Jiwakanon J Pringle M Fernström L-L. Antimicrobial use and antimicrobial susceptibility in *Escherichia coli* on small- and medium-scale pig farms in north-eastern Thailand. Antimicrob Resist Infect Control. (2017) 6:75. 10.1186/s13756-017-0233-9 28725421 PMC5512823

[10] Visschers VHM Backhans A Collineau L Iten D Loesken S Postma M. Perceptions of antimicrobial usage, antimicrobial resistance and policy measures to reduce antimicrobial usage in convenient samples of Belgian, French, German, Swedish and Swiss pig farmers. Prev Vet Med. (2015) 119:10–20. 10.1016/j.prevetmed.2015.01.018o 25684036

[11] Dau NH Kháng sinh trong chăn nuôi, tôn du kháng sinh, kháng sinh và giai pháp phòng chòng thích họp. In: Proceedings of National Animal Science and Veterinary Medicine Conference (Can Tho). (2017) pp. 75–85.

[12] Catley A Alders RG Wood JLN. Participatory epidemiology: approaches, methods, experiences. Vet J. (2012) 191:151–60. 10.1016/j.tvjl.2011.03.010 21856195

[13] Mariner J Paskin R FAO Animal Health Manual 10 Manual on Participatory Epidemiology Method for the Collection of Action-Oriented Epidemiological Intelligence. Food and Agriculture Organization Rome (2000).

[14] Brown SR Political Subjectivity: Applications of Q Methodology in Political Science. New Haven, CT: Yale University Press (1980).

[15] Farrimond H Joffe H Stenner P. A Q-methodological study of smoking identities. Psychol Health. (2010) 25:979–98. 10.1080/08870440903151080 20309778

[16] Garner ID A q-methodological study of male attitudes towards testicular cancer and testicular self-examination. Inquiries Journal/Student Pulse, 3 (2011). Retrieved from: http://www.inquiriesjournal.com/a?id=592

[17] Truong DB Binot A Peyre M Nguyen NH Bertagnoli S Goutard FL. A Q method approach to evaluating farmers' perceptions of foot-and-mouth disease vaccination in vietnam. Front Vet Sci. (2017) 4:95. 10.3389/fvets.2017.00095 28695123 PMC5483627

[18] Previte J Pini B Haslam-McKenzie F Q methodology and rural research. Sociol Rural. (2007) 47:135–47. 10.1111/j.1467-9523.2007.00433.x

[19] Danielson S Webler T Tuler SP Using Q method for the formative evaluation of public participation processes. Soc Nat Resour. (2009) 23:92–6. 10.1080/08941920802438626

[20] Guest G Namey E McKenna K How many focus groups are enough? building an evidence base for nonprobability sample sizes. Field Methods. (2017) 29:3–22. 10.1177/1525822X16639015

[21] Ameri AA Hendrickx S Jones B Mariner J Mehta P Pissang C Introduction to Participatory Epidemiology and Its Application to Highly Pathogenic Avian Influenza Participatory Disease Surveillance: A Manual for Participatory Disease Surveillance Practitioners. (2009). Available online at: https://cgspace.cgiar.org/handle/10568/367 (Accessed September 26, 2016).

[22] Bryant LD Burkinshaw P House AO West RM Ward V Good practice or positive action? Using Q methodology to identify competing views on improving gender equality in academic medicine. BMJ Open. (2017) 7:e015973 10.1136/bmjopen-2017-015973 PMC5629690 28830870

[23] Van Exel J De Graaf G Q methodology: a sneak preview. (2005). Available online at: https://qmethodblog.files.wordpress.com/2016/01/qmethodologyasneakpreviewreferenceupdate.pdf

[24] Husson F Lê S Pagès J Exploratory Multivariate Analysis by Example Using R. Boca Raton, FL: CRC Press (2011).

[25] Zabala A qmethod: A Package to Explore Human Perspectives Using Q Methodology. (2014). Available online at: https://www.repository.cam.ac.uk/handle/1810/248225 (Accessed February 24, 2017).

[26] Leggette HR Redwine T Using Q methodology in agricultural communications research: a philosophical study. J Appl Commun. (2016) 100:57–67. 10.4148/1051-0834.1230

[27] Watts S Stenner P Doing Q ethodology: theory, method and interpretation. Qual Res Psychol. (2005) 2:67–91. 10.1191/1478088705qp022oa

[28] Huang R RQDA: R-Based Qualitative Data Analysis. R package version 0.2-8 (2016). Available online at: http://rqda.r-forge.r-project.org/

[29] R Core Team. R: A Language and Environment for Statistical Computing. Vienna: R Foundation for Statistical Computing (2017). Available online at: http://www.R-project.org/

[30] Rushton J. The Economics of Animal Health and Production. First paperback edition. Wallingford; Cambridge, MA: CABI (2011).

[31] Graham JP Eisenberg JNS Trueba G Zhang L Johnson TJ Small-scale food animal production and antimicrobial resistance: mountain, molehill, or something in-between? Environ Health Perspect. (2017) 125:104501 10.1289/EHP2116 29038091 PMC5933306

[32] Cuong NV Truc VNT Nhung NT Thanh TT Chieu TTB Hieu TQ. Highly pathogenic avian influenza virus A/H5N1 infection in vaccinated meat duck flocks in the Mekong Delta of Vietnam. Transbound Emerg Dis. (2016) 63:127–35. 10.1111/tbed.12470 26748550 PMC4819680

