# Supplementary material for: Assessment of Drivers of Antimicrobial Usage in Poultry Farms in the Mekong Delta of Vietnam: A Combined Participatory Epidemiology and Q-Sorting Approach
Source: Front Vet Sci. 2019 Mar 25;6:84. doi: 10.3389/fvets.2019.00084 (PMC6442645; doi:10.3389/fvets.2019.00084)
Supplement: Supplementary 1 — Checklist used for CI-participatory epidemiology approach. [file Table_1.DOCX]

Supplementary 1. Checklist used for collective interview discussion - participatory epidemiology approach.

1. Characterization of the diseases perceived as important by producers (clinical and epidemiological description), and associated prevention and control strategies for each disease mentioned by farmers.

- List the important diseases in poultry production; describe them by the clinical and epidemiological features.

- Pairwise ranking the important diseases (limit in 5).

- List at least 2 prevention and control methods for each disease mentioned above (pay attention on disease using antimicrobial for latter question).

2. Identify moment of using antimicrobial during production cycle (limit at 5 first month of cycle)

- Timeline and proportional pilling exercise

3. Identification source of advice and procurement of antimicrobials to farmers

- List of actors contacted by farmers

- Proportional pilling exercise for scoring important level of each source of advice.

- Probing question on the most and the less important sources.

4. Identification positive and negative aspects of AMU.

- Flow chart
