# Supplementary material for: Assessment of Drivers of Antimicrobial Usage in Poultry Farms in the Mekong Delta of Vietnam: A Combined Participatory Epidemiology and Q-Sorting Approach
Source: Front Vet Sci. 2019 Mar 25;6:84. doi: 10.3389/fvets.2019.00084 (PMC6442645; doi:10.3389/fvets.2019.00084)
Supplement: Supplementary 2 — Script for Q-sorting interviews analysis run in R program. [file Table_2.DOCX]

# Q-sorting analysis

#Code prepared by Bao, Phu

rm(list = ls())# clean all of dataset that automatically remembered by R

#Read dataset

setwd("C:/Users/baotd/Desktop")

d_Q <- read.csv2("Q sorting data.csv",sep=",", na.strings = " ", header=TRUE)

d_Q # review data

d_Q<- data.frame(d_Q)

d_Q <- d_Q[,-c(1)]

# Principal Component Analysis (PCA)

d_Q_farmer<-d_Q[,c(1:28)]# dataset for farmer's group

d_Q_vet<-d_Q[,c(29:54)]# dataset for advisor's group

# Step 1:Analyse and and compaire distribution (min, max, moyenne, variance, etc.)

# Perform PCA using package FactomineR

library(FactoMineR)

res.pca.testQ_f <-PCA(d_Q_farmer)

res.pca.testQ_a <-PCA(d_Q_vet)

summary(res.pca.testQ_f)

summary(res.pca.testQ_a)

#calculation eigenvalue for farmer's group and advisor's group

res.pca.testQ_f$eig

res.pca.testQ_a$eig

# plot of the eigenvalues

barplot(res.pca.testQ_f$eig[,1],main="Eigenvalues",names.arg=1:nrow(res.pca.testQ_f$eig),xlab=NULL,

ylab="Percentage of variance")

barplot(res.pca.testQ_a$eig[,1],main="Eigenvalues",names.arg=1:nrow(res.pca.testQ_a$eig),xlab=NULL,

ylab="Percentage of variance")

# Factor analysis with "qmethod" package

library(qmethod)

# Farmer's group, number of factor =4

results.testQ_d_Q_farmer4 <- qmethod(d_Q_farmer, nfactors=4, rotation="varimax")

summary(results.testQ_d_Q_farmer4)

title <- "Q method z-scores, d_Q dataset"

subtitle <- paste0("Four factors_farmer, PCA, varimax. Printed on ",

Sys.Date())

x11()

plot(results.testQ_d_Q_farmer4, main=title, sub=subtitle,cex.main=1.8,cex.lab=1.2)

print(results.testQ_d_Q_farmer4, length=NULL, digits=1)

# Advisor's group, number of factor =3

#case1: number of factors=3

results.testQ_d_Q_vet3 <- qmethod(d_Q_vet, nfactors=3, rotation="varimax")

summary(results.testQ_d_Q_vet3)

title <- "Q method z-scores, d_Q dataset"

subtitle <- paste0("Three factors_vet, PCA, varimax. Printed on ",

Sys.Date())

x11()

plot(results.testQ_d_Q_vet3, main=title, sub=subtitle,cex.main=1.8,cex.lab=1.2)

print(results.testQ_d_Q_vet3, length=NULL, digits=1) # length =...: number of statement that will be present

#######################End###################
