# Supplementary figures and images for: Assessment of Drivers of Antimicrobial Usage in Poultry Farms in the Mekong Delta of Vietnam: A Combined Participatory Epidemiology and Q-Sorting Approach
Source: Front Vet Sci. 2019 Mar 25;6:84. doi: 10.3389/fvets.2019.00084 (PMC6442645; doi:10.3389/fvets.2019.00084)

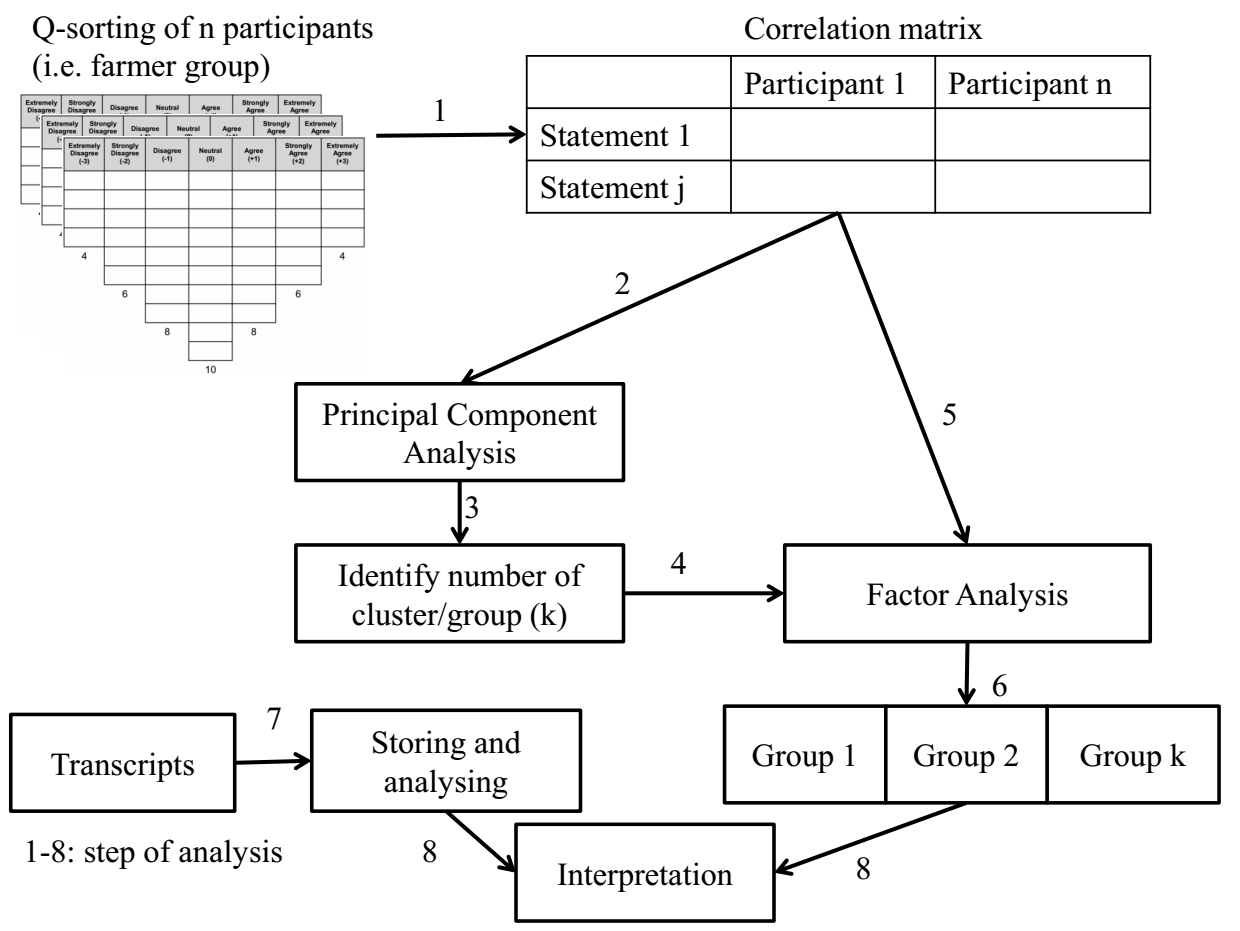

Supplement: Supplementary Figure 1 — Flow chart of study steps. [file Image_1.TIF]
