# Supplementary material for: Assessment of Drivers of Antimicrobial Usage in Poultry Farms in the Mekong Delta of Vietnam: A Combined Participatory Epidemiology and Q-Sorting Approach
Source: Front Vet Sci. 2019 Mar 25;6:84. doi: 10.3389/fvets.2019.00084 (PMC6442645; doi:10.3389/fvets.2019.00084)
Supplement: Supplementary Figure 2 — Flow chart of statistical analyses steps. [file Data_Sheet_2.PDF]

(1) Collection and summary of descriptive data using PE tools (4 themes)

Planned: 25 CI, 5 stakeholders, (250 participants)

Actual: 26 CI, 5 stakeholders, (198 participants)

(a) Characterise diseases in poultry farms, and describe farmers' strategies to prevent and control them
(b) Identify the timing of AMU in relation to the amounts used
(c) Identify sources of advice and procurement of antimicrobials to farmers
(d) Identify opinions about positive and negative aspects of AMU

(2) Generation of statements

46 statements, 4 thematic areas

(a) Farmers' reliance on antimicrobials to prevent and treat disease
(b) Logistics of AMU in the field
(c) Costs of antimicrobials
(d) Impact of AMU on animal productivity and AMR.

(3) Participant selection

(60 participants)

Maximise diversity (based on type of production, gender, age, experience, level of education)

(4) Individual interviews for Q-sorting

(60 participants)

Ranking of statements according to level of agreement/disagreement

(5) Q-sorting analysis

(**54 participants, 28 farmers and 26 advisors**)

Exclude individuals providing incomplete data (6 participants)
